# Endermol, a New Vehicle for Ointments

**Published:** 1904-10

**Authors:** 


### ABSTRACTS.

### Endermol, a New Vehicle for Ointments.

Virgil Coblentz, New York, (*Medical News,* September 3, 1904,) says that endermol, a combination of stearic acid amide and paraffin hydrocarbons, forms an almost white mass of about the consistency of lard, inodorous, of neutral reaction and fusing at 78° to 80°C. The iodine absorption number is 16.98, while that of lard averages about 62; a point in favor of the former as a vehicle for iodine.

When exposed to the air and sunlight under adverse conditions, samples of endermol retained their color, consistency and blandness. When applied to the skin by inunction, endermol forms a smooth, soft, unctuous mass which is readily absorbed. This may be demonstrated comparatively by applying a little of petrolatum, lanolin and endermol in separate portions by inunction to the skin and noting the time required for absorption. Furthermore, applications of endermol ointments of iodine (without potassium iodide), and also of aconite were followed by the excretion of iodine in the urine after about five hours in the former, and the characteristic dryness of the throat in the latter.

Rancidity in fats is due to hydrolysis or splitting up of the esters of the fatty acids, with liberation of the free acids. To the presence of these the irritating properties of rancid fats are due. In endermol, consisting chiefly of stearic amide, we have a stable fatty acid derivative which is not decomposed under any conditions through the action of light, air, moisture or such chemicals as are usually employed in ointments.

To demonstrate the adaptability of endermol as a vehicle for ointments, mechanically as well as chemically, ointments with such substances as the yellow and red mercuric oxide, yellow and red iodide of mercury, zinc oxide, lead subacetate and carbonate, ichthyol, tar, vegetable extracts and mercury were prepared. All of these yielded smooth, uniform ointments which showed no change whatever upon exposure.

The use of petrolatum, as is well known, is restricted to that of a dressing and this to a limited extent owing to its immiscibility with water, certain chemicals and galenicals.

Lanolin, an excellent vehicle, is objectionable because of its stickiness and toughness; and when combined with animal fats (as in lanolin cream), such ointments become rancid and offensive.

There is nothing so objectionable to either the physician or pharmacist as a rancid ointment. While lard or other animal fats may originally have been anhydrous and benzoinated, yet rancidity ensues sooner or later, particularly so when aqueous fluids have been incorporated, as frequently is the case in ointments. Aside from the irritant action, chemical reaction of the liberated fatty acids upon the medicinal agent follows.

Again, lard substitutes consisting of mixtures of suet or tallow or cotton-seed oil, stearin with cotton seed, sesame or cocoanut oils, are not only open to the same objections, but also the possible presence of alum, alkalies or water, which render them still less desirable.

Because of its blandness, pliability and freedom from stickiness, endermol is especially adapted as a lubricant for massage treatment, always leaving the skin soft and pliable.

To sum up, the advantages of endermol as an ointment vehicle are:

Absolute freedom from any tendency towards rancidity, although as much as 15 per cent. of water may be incorporated.

Ready penetrability and absorption.

Pliability, smoothness and freedom from stickiness.

Freedom from irritating properties.

